# Venom Immunotherapy Does Not Affect Survival of Patients with Malignant Tumor in Poland

**DOI:** 10.3390/jcm13113152

**Published:** 2024-05-28

**Authors:** Marta Chełmińska, Krzysztof Specjalski, Ewa Jassem, Joanna Polańska, Karolina Kita, Lucyna Górska, Joanna Didkowska, Urszula Wojciechowska, Marita Nittner-Marszalska, Piotr Kuna, Maciej Kupczyk, Jerzy Kruszewski, Aleksander Zakrzewski, Ewa Czarnobilska, Marcin Stobiecki, Rafał Krenke, Andrzej Dąbrowski, Artur Kwaśniewski, Jerzy Jarząb, Andrzej Bożek, Anna Bodzenta-Łukaszyk, Mateusz Łukaszyk, Marek Kowalski, Ewa Smorawska-Sabanty, Andrzej Fal, Katarzyna Przybyłowska, Zbigniew Bartuzi, Krzysztof Pałgan, Marek Niedoszytko

**Affiliations:** 1 Department of Allergology, Medical University of Gdańsk, 80-952 Gdańsk, Poland; allergy@gumed.edu.pl (M.C.); karolina.kita@gumed.edu.pl (K.K.); lucyna.gorska@gumed.edu.pl (L.G.); mnied@gumed.edu.pl (M.N.); 2 Department of Pneumonology, Medical University of Gdańsk, 80-952 Gdańsk, Poland; ejassem@gumed.edu.pl; 3 Department of Data Science and Engineering, Silesian University of Technology, 44-100 Gliwice, Poland; joanna.polanska@polsl.pl; 4 National Institute of Oncology, 00-001 Warsaw, Poland; didkowskaj@coi.waw.pl (J.D.); wojciechowskau@coi.waw.pl (U.W.); 5 Department of Internal Diseases, Pneumonology and Allergology, Medical University of Wroclaw, 50-368 Wroclaw, Poland; marita.nittner-marszalska@umw.edu.pl; 6 Department of Internal Diseases, Asthma and Allergy, Medical University of Łódź, 90-419 Łódż, Poland; piotr.kuna@umed.lodz.pl (P.K.); maciej.kupczyk@umed.lodz.pl (M.K.); 7 Department of Infectious Diseases and Allergology, Military Institute of Medicine, 04-141 Warsaw, Poland; j.kruszewski@hipokrates.org (J.K.); olozak@wp.pl (A.Z.); 8 Centre of Clinical and Environmental Allergology, Jagiellonian University, 31-503 Cracow, Poland; ewa.czarnobilska@uj.edu.pl (E.C.); marcin.stobiecki@uj.edu.pl (M.S.); 9 Department of Internal Diseases, Pneumonology and Allergology, Medical University of Warsaw, 02-097 Warsaw, Poland; rafal.krenke@wum.edu.pl (R.K.); andrzej.dabrowski@wum.edu.pl (A.D.); 10 Hospital CDT Medicus Sp. z o. o., 59-300 Lubin, Poland; kwasniewski@cdtmedicus.pl; 11 Clinical Department of Internal Medicine, Dermatology and Allergology, Medical University of Silesia, 40-055 Zabrze, Poland; jerzy.jarzab@gmail.com (J.J.); andrzejbozek@o2.pl (A.B.); 12 Department of Allergology and Internal Diseases, University Hospital, 15-089 Białystok, Poland; abodzentalukaszyk@gmail.com; 13 I Department of Pulmonary Diseases and Tuberculosis, Medical University of Białystok, 15-089 Białystok, Poland; mlukaszyk@gmail.com; 14 Department of Immunology, Reumatology and Allergy, Medical University of Łódź, 92-213 Łódź, Polandewasm@csk.umed.lodz.pl (E.S.-S.); 15 Department of Allergology, Pulmonary Diseases and Internal Medicine, MSWiA Central Clinical Hospital, 02-507 Warsaw, Poland; amfal@wp.pl (A.F.); przybylowskakasia@gmail.com (K.P.); 16 Department of Allergology, Clinical Immunology and Internal Diseases, Jan Biziel University Hospital, Nicolaus Copernicus University, 85-067 Bydgoszcz, Poland; zbartuzi@cm.umk.pl (Z.B.); palgank@wp.pl (K.P.)

**Keywords:** allergy and cancer, insect venom allergy, immunotherapy safety, venom immunotherapy

## Abstract

**Background:** Allergen immunotherapy (AIT) is a well-established and efficient method of causative treatment for allergic rhinitis, asthma and insect venom allergy. Traditionally, a recent history of malignant neoplasm is regarded as a contraindication to AIT due to concerns that AIT might stimulate tumor growth. However, there are no data confirming that the silencing of the Th2 response affects prognosis in cancer. **Objectives:** The aim of this study was to investigate frequency of malignant tumors in patients undergoing AIT and the association between AIT and cancer-related mortality. **Patients and Methods:** A group of 2577 patients with insect venom allergy undergoing AIT in 10 Polish allergology centers was screened in the Polish National Cancer Registry. Data on cancer type, diagnosis time and patients’ survival were collected and compared with the general population. **Results:** In the study group, 86 cases of malignancies were found in 85 patients (3.3% of the group). The most common were breast (19 cases), lung (9 cases), skin (8 cases), colon and prostate cancers (5 cases each). There were 21 cases diagnosed before AIT, 38 during and 27 after completing AIT. Laplace’s crude incidence rate was 159.5/100,000/year (general population rate: 260/100,000/year). During follow-up, 13 deaths related to cancer were revealed (15% of patients with cancer). Laplace’s cancer mortality rate was 37.3/100,000/year (general population rate: 136.8/100,000/year). **Conclusions:** Malignancy was found in patients undergoing immunotherapy less often than in the general population. Patients with cancer diagnosed during or after AIT did not show a lower survival rate, which suggests that AIT does not affect the prognosis.

## 1. Introduction

*Hymenoptera* venom allergy manifested by stinging-related anaphylaxis is reported in 1–3% of the population [1]. Risk of severe systemic reaction after stinging is 5–15% in patients with a history of large local reaction, 14–20% after mild systemic reaction (grades I–II in Mueller’s classification) and approximately 80% after severe systemic reaction (grades III–IV). Thus, the most significant risk factor of stinging-related anaphylaxis is a history of severe reaction. Another risk factors include older age, concomitant mast cells disorders, cardiovascular diseases, bee venom allergy and frequent stinging [2,3].

According to EAACI guidelines, all patients with a history of stinging-related anaphylaxis should be advised to avoid activities associated with increased risk of stinging and be equipped with an autoinjector with adrenaline [4]. However, the only causative treatment is venom immunotherapy (VIT). It leads to long-term tolerance of venom allergens and substantially decreases risk of future anaphylaxis. Venom immunotherapy is regarded as a safe procedure, efficiently protecting against anaphylaxis in more than 80% of patients on bee venom immunotherapy, and more than 90% of those on wasp venom immunotherapy [5,6]. 

In clinical practice there is only a limited number of absolute or relative contraindications to VIT [7]. Traditionally, one of the clinical contraindications to immunotherapy is a history of malignant neoplasm, due to concerns that VIT might stimulate tumor growth or affect immunological surveillance. In asthma and allergic rhinitis, cancer is regarded as an absolute contraindication to immunotherapy, while in insect venom allergy it is relative. However, this approach is highly speculative as there are no convincing data confirming that silencing of the Th2 response during VIT affects the efficacy of cancer treatment or has an unfavorable effect on tumor growth. Moreover, in high-risk venom allergic patients with remission of malignancy, postponing VIT could have deleterious consequences and risk of stinging-related death seems to outweigh possible risks associated with VIT.

### Aim of the Study

The aim of this study was to investigate frequency of malignant tumors in patients undergoing venom immunotherapy, as well as the association between VIT and cancer-related mortality.

## 2. Materials and Methods

1. Study group

The study group involved 3102 adult patients with insect venom allergy who underwent VIT between 1998 and 2019. All the participants had a history of stinging-related anaphylaxis. Sensitization to wasp or bee venom had been confirmed by means of skin testing and/or serum-specific IgE. Exclusion criteria included clinically relevant respiratory or cardiovascular insufficiency, current infection, active autoimmune disease, pregnancy, active cancer or poor compliance [4].

The group was recruited in 12 Polish allergology centers in 9 cities (Table 1).

2. Protocol of the study

Insect venom immunotherapy was conducted in accordance with EAACI guidelines [4]. Briefly, during the initial phase, tolerance of 100 μg of venom was induced using *rush* or *ultra rush* protocols. Maintenance doses of 100 μg of venom were administered over five consecutive years every four to eight weeks. VIT was prolonged in cases of accompanying mast cell disorders. As soon as any of the mentioned above contraindication was revealed and found relevant in the assessment of an investigator, the treatment was ceased prematurely.

In 2019, having anonymized the files, we cross-referenced patients’ data with the Polish Cancer Registry to find information about the occurrence of cancer. In cases of cancer, we additionally obtained data on the type of cancer and date of diagnosis, including the time between diagnosis and immunotherapy. At the end of the three-year observation period (autumn 2022), the vital status of patients previously diagnosed with cancer was additionally collected.

In statistical analysis, data on the incidence of malignancies and cancer-related mortality rate were compared with the general population.

The protocol of the study was approved by the Independent Bioethics Committee at Medical University of Gdansk (NKBBN/256/2014; approval date: 8 July 2014). 

## 3. Results

1. Incidence of malignancy in the study group

Out of 3102 participants enrolled, complete clinical data were acquired for 2545 patients, and only this group was involved in further analysis. 

Screening in the Polish National Cancer Registry revealed 86 cases of malignancy in 85 patients (3.3% of the group), including 67 participants on immunotherapy with wasp venom, 16 with bee venom and 2 with both. Detailed clinical data on patients diagnosed with malignancy have been presented in Table 2. 

**Figure 1:**
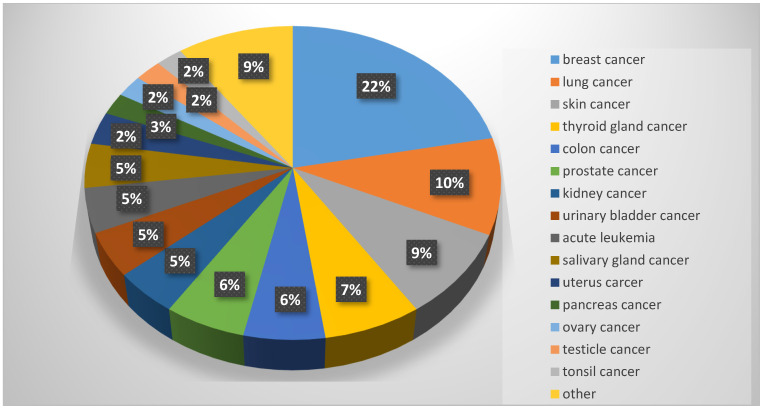
Incidence of neoplasms in the study group (n = 2545 participants; 86 cases of malignancy).

Overall, Laplace’s crude cancer incidence rate in study participants was 159.5/100,000/year (incidence in general Polish population: 260/100,000/year). The most common types of malignancies in our group were breast cancer (19 cases), lung cancer (9 cases), skin cancer (8 cases) and thyroid gland cancer (6 cases), as shown in Figure 1. Incidence rates for the most common malignancies, as well as corresponding data for the general Polish population, are presented in Table 3. The study group was characterized by lower incidence of every malignancy compared to the general adult population. 

The mean age at cancer diagnosis was 57 years. Nevertheless, the group was quite diverse in this respect, with several tendencies related to cancer type. Lung and intestinal cancer were diagnosed more often in older patients (67 and 65 years, respectively) compared to breast cancer or thyroid gland cancer (54 and 43 years, respectively). 

In our study group, 21 cases of malignancies were diagnosed before VIT was initiated. The interval between cancer diagnosis and VIT was between 9 months and 28 years (mean interval: 28 months). The most common malignancies in this subgroup were breast cancer (six cases) and urinary bladder cancer (three cases). 

In 38 cases, cancer was diagnosed during VIT (i.e., 3 months to 13 years after initiating VIT; mean duration of VIT until cancer diagnosis: 15 months). The most common malignancies in this subgroup were breast, thyroid gland and lung cancers, found in 12, 4 and 4 patients, respectively. 

Finally, 27 cases were diagnosed having completed VIT. Intervals between termination of VIT and diagnosis of cancer were between 2 weeks and 8.5 years (mean interval: 10 months). The most common in this subgroup were skin cancer (four cases), lung cancer (four cases) and prostate cancer (three cases).

2. Cancer-related mortality in the study group

During follow-up, 13 cancer-related deaths were revealed, equivalent to 15% of patients diagnosed with cancer. Laplace’s cancer mortality rate was 37.3/100,000/year. By contrast, the cancer mortality rate in the general Polish population is 136.8/100,000/year.

In the subgroup of 21 patients diagnosed with cancer before immunotherapy, two cases of relapses leading to death were found (9.5% of the subgroup), both resulting from bladder cancer. The first patient was diagnosed with cancer in 2003. Having completed five-year observation, he started VIT in 2008 and therapy was continued until 2012. The patient died because of cancer relapse in 2019, i.e., seven years after termination of VIT. The second patient was diagnosed with bladder cancer in 2002. VIT was initiated in 2010 and continued for a year. The patient died in 2018, i.e., eight years after termination of VIT.

In the subgroup with cancer diagnosed during VIT, there were five fatal cases, including two patients with lung cancer and one with gallbladder, hepatic and cervical cancers. 

Finally, six deaths were noted in patients diagnosed after termination of VIT. Two cases were related to acute myeloid leukemia and one with each of the following: myeloma multiplex, melanoma, cervical cancer and disseminated cancer of unknown primary location. Mean interval between termination of VIT and death was 66 months (20–127 months).

## 4. Discussion

Allergen-specific immunotherapy (AIT) is a recommended causative method of treatment of IgE-mediated allergic diseases, restoring normal immunity against allergens. Immunotherapy modulates both cellular and humoral responses. Its early consequences stem from mast cell and basophil desensitization. Intermediate effects are related to several changes in allergen-specific T cells and late effects are associated with decreased IgE synthesis and an altered function of mast cells, basophils and eosinophils. However, it is generally believed that the key phenomenon in the tolerance induction is Th2 cell suppression by Treg cells secreting IL-10 and TGFβ. IL-10 has a suppressive effect on the effector cells (mast cells, basophils) and changes the type of humoral response from an IgE- to IgG_4_-dominated phenotype [9]. The early phase of immunotherapy is associated with a transient increase of serum-specific IgE [10]. However, it does not lead to enhanced reactivity to allergens upon natural exposure nor treatment-related unwanted effects. On the contrary, the elevation of IgE in early stages of AIT stems from Th2 cells priming with high doses of allergens and may be associated with better response in the future. In the maintenance phase of AIT, a gradual decrease of specific IgE is observed [11,12]. As far as IgG is concerned, during AIT the levels increase significantly, even 100-fold, particularly in regard to the IgG_4_ class [12,13]. This effect correlates with the duration of AIT and the cumulative dose of allergen used [14]. Several publications revealed the inhibitory effect of IgG_4_ in relation to IgE-dependent reactions [15]. IgG_4_ bind allergens, preventing their binding with IgE and, in this way, degranulation of mast cells. Although serum concentrations of IgG and IgG_4_ fall significantly soon after termination of immunotherapy, the beneficial effect of AIT persists for years [14].

All the mechanisms playing a role in regaining tolerance during immunotherapy also participate in the regulation of the immune response to cancer. Both IL-10 and TGF-β have a pivotal role in the establishment of tolerance to allergens but can also be secreted by cancer cells. Their production in a tumor correlates with the disease’s progression [16]. Tregs, a source of IL-10, suppress allergies, but their accumulation in cancer tissue has been found to be associated with the disease progression. IgG_4_ are considered anti-inflammatory antibodies playing a protective role in parasitic infestation and allergies. As tumors are characterized by chronic inflammation with long-term exposure to cancer antigens, high concentrations of IgG_4_ were detected in several malignancies, including pancreatic cancer, cholangiocarcinoma, glioblastoma and melanoma [17]. IgG_4_ overexpression correlates with tumor progression [18]. IgE has a surveillance function in the cancer. It has been demonstrated that solid tumors are infiltrated by IgE receptor-expressing immune cells and anti-tumor IgE may result in antibody-dependent cell-mediated cytotoxicity and phagocytosis of cancer cells [19]. Conversely, IgE deficiency is a susceptibility factor for cancer [20].

As several cytokines and cell types crucial for immunologic tolerance are also involved in cancer surveillance, several safety concerns were raised in relation to initiating or continuing AIT in patients recently diagnosed with malignant neoplasm. As a consequence, according to the EAACI guidelines, AIT with airborne allergens is absolutely contraindicated in patients with cancer and VIT is relatively contraindicated [7]. This approach is shared by a large number of national allergology societies [21].

Clinical evidence for these recommendations is not sufficient, as reports on the prognosis in patients with a history of cancer who initiated or continued AIT are scarce. In 2011, Worhl et al. reported a series of six cases of patients suffering or having suffered from malignant neoplasia (four melanomas, one lung cancer and one breast cancer) and concomitant IgE-mediated allergy. Despite the history of cancer, four patients continued insect venom immunotherapy and one with pollens, with no cancer relapse [22]. On the other hand, Brożek et al. attempted to determine the prevalence of neoplasms during long-term follow-up of 1144 patients who had received immunotherapy with house dust mites or pollens due to asthma or allergic rhinitis. Over the following 20 years, cancer incidence in the study group did not differ significantly from the control group. Interestingly, there was an inverse association between immunotherapy and the prevalence of new chronic myeloid leukemia and chronic lymphoblastic leukemia [23]. In the Danish population, it was demonstrated that allergic patients treated with immunotherapy were characterized by lower overall mortality compared to a group receiving conventional allergy treatment [24]. What is more, IgE-mediated allergy was not found to either be protective against cancer or increase its incidence [25].

In Poland, cancer leads to nearly 100,000 deaths each year, which is 25% of all deaths (Table 3). Thus, it is the second most significant cause of mortality just after cardiovascular diseases. It is estimated that approximately 1.17 million Polish citizens struggle with cancer currently, and the incidence of new cases is approximately 171,000 per year. In women, the most common malignancies include lung, breast and colon cancers, while in men they are prostate, lung and colon cancers [8]. 

Our study included patients with insect venom allergy and a history of stinging-related anaphylaxis. In this population, immunotherapy can be regarded as a life-saving treatment due to the fact that it decreases the risk of future stinging-related anaphylaxis. As a consequence, postponing VIT because of oncologic safety concerns may paradoxically increase mortality risk associated with anaphylaxis, particularly in patients with high risk of stinging (beekeepers, gardeners, etc.). 

We have demonstrated that the prevalence of new cases or relapses of already diagnosed malignancy in the group undergoing VIT is lower compared to the general population. This is in line with the previous study of Brożek et al. and corresponds well with the reports suggesting that cancer is not regarded by allergologists as a frequent problem in conducting immunotherapy in clinical practice [23,26]. However, we are aware of possible bias in our study. First of all, the prevalence of cancer increases in the last decades of life, due to impaired immunologic surveillance, cumulation of exposure to cancerogenic factors, etc. This part of the population is characterized by the presence of several chronic diseases and impaired functioning. Thus, it is always underrepresented in studies on immunotherapy. Some of the conditions common in this age group (unstable cardiac insufficiency, respiratory insufficiency) are contraindications to VIT. Others, such as mobility limitation or memory impairment, are practical obstacles to undergoing VIT. As a result, elderly patients are less willing to initiate five-year treatment associated with regular visits to an allergology center. On the other hand, health-conscious and younger members of local communities with better access to healthcare, rarely diagnosed with cancer, are more prone to undergo VIT. 

The significant limitation of our study is the fact that we only had full clinical data from patients who developed cancer. Thus, we were not able to compare patients on VIT with and without cancer history in terms of cancer risk factors, lifestyle, concomitant diseases, etc. Carcinogenesis has been demonstrated to be a multifactorial process affected by patient’s genetic background, lifestyle and exposure to carcinogenic substances. The condition of the immune system is only one of the significant regulators of carcinogenesis. It could be speculated that patients on VIT are more health-oriented and the lower prevalence of cancer in our group may result from their lifestyle rather than immunotherapy itself. 

Secondly, patients enrolled in the study were not characterized by higher cancer-related mortality. In cases of previously diagnosed cancer, relapse resulting in death was found in 9.5% of the subgroup. Both cases were associated with bladder cancer. European data for bladder cancer indicate that the average five-year survival rate is 68.8%, while in Polish patients after cystectomy, the five-year survival rate is only 32% [27,28]. Among 65 patients with cancer during/after VIT, 11 fatal cases were revealed (17% of the group). Data from the Polish Registry of Cancer demonstrated that in patients diagnosed with cancer between 1999 and 2010, five-year survival was 41.3% in men and 56.1% in women [29]. Thus, our results seem to suggest that VIT is not associated with any significant impairment of the immune response towards, or lower efficacy of, oncologic treatment.

Safety is a key feature of any therapy. In chronic allergic diseases, treatment is usually long-term and often modulates the function of the immune system, affecting anti-microbial or anti-parasitic responses and tumor growth surveillance. In recent years, biologic treatment has become a significant part of the therapy of severe asthma, chronic urticaria, chronic sinusitis and atopic dermatitis [30,31]. Currently used biologicals are antibodies against several molecules, mostly involved in T2-high inflammation (IL-5, IL-5R, IL-4R, IgE). Initially, their introduction raised concerns about possible delayed complications associated with immunomodulation. In recent years these concerns have not been confirmed. However, due to the novelty of these therapies, we still lack data on prolonged observations. 

On the other hand, allergen immunotherapy is well-known and has been widely used for decades. However, only after dynamic development of immunology was it possible to discover that tolerance induction and cancer surveillance share several mechanisms. Cancer is a multifactorial group of pathologies of diverse distribution depending on genetic background, lifestyle and exposure to carcinogenic factors. Thus, relations between VIT and cancer development may be varied and differ between populations of diverse ethnicity, social and economic backgrounds. Future studies should address this issue in multivariate analyses attempting to determine relations between VIT, lifestyle and cancer prevalence. Prolonged monitoring of study participants seems crucial, as cancer is usually the delayed consequence of interactions between genes and environment and, as a result, is most commonly diagnosed in the elderly. 

## 5. Conclusions

Insect venom immunotherapy is not associated with higher incidence of malignancy. In patients with insect venom allergy who developed cancer, the mortality rate is lower compared to the general population with cancer. 

## Figures and Tables

**Table 1:** Number of study participants and cancer cases in distinct cities.

City	Number of Patients Included	Number of Cancer Cases	% of Patients with Cancer History
Białystok	85	2	2.3%
Bydgoszcz	525	16	3.0%
Gdańsk	651	37	5.6%
Kraków	187	3	1.6%
Lubin	125	4	3.2%
Łódź	457	18	3.9%
Warszawa	393	21	5.3% *
Wrocław	573	10	1.7%
Zabrze	106	2	1.9%

* In Warsaw one of the patients had two independent episodes of malignancy.

**Table 2:** Clinical characteristics of patients undergoing VIT who had an episode of cancer.

Characteristic of the Study Participants	
Gender	Women: 55 Men: 30
Age at cancer screening	20–94 years (mean: 65 years)
Age at diagnosis of cancer	16–87 years (mean: 57 years)
Allergen used for VIT	Wasp venom: 67 patients Bee venom: 16 patients Wasp and bee venom: 2 patients
Grade of stinging-related anaphylaxis (Mueller’s scale)	I 1 patient II 3 patients III 52 patients IV 29 patients
Duration of VIT	4–154 months (mean: 49 months)
Cumulative dose of vaccine given on VIT	411–6300 μg (mean: 3676 μg)
Vaccines applied for initial phase of VIT	Pharmalgen: 33 patients Venomenhal: 52 patients
Vaccines applied for maintenance phase of VIT	Pharmalgen: 2 patients Venomenhal: 39 patients Alutard: 44 patients

**Table 3:** Cancer incidence and mortality in the general Polish population between 2000 and 2020 and in the study group. Data derived from Polish National Registry of Cancer [8].

	Data for General Polish Population			Study Group 2019
Year	2000	2010	2020	
Total cancer incidence (no. new cases)	119,225	145,822	147,877	
Total cancer incidence—crude rate (per 100,000)	311.65	378.59	385.56	159.5
Lung cancer incidence (no. new cases)	19,989	21,384	18,997	
Lung cancer incidence—crude rate (per 100,000)	52.25	55.52	49.53	16.84
Breast cancer incidence (no. new cases)	12,664	16,300	17,807	
Breast cancer incidence—crude rate (per 100,000)	33.10	42.32	46.43	37.33
Colon cancer incidence (no. new cases)	6443	9452	9444	
Colon cancer incidence—crude rate (per 100,000)	16.84	24.54	24.62	9.35
Total cancer mortality (no. cases)	84,559	92,605	99,870	
Total cancer mortality—crude rate (per 100,000)	221.03	240.43	260.39	37.3

## Data Availability

All data available on demand.

## Abbreviations

AIT	allergen immunotherapy
EAACI	European Academy of Allergy and Clinical Immunology
TGFβ	transforming growth factor beta
VIT	venom immunotherapy
IL-4R	interleukin 4 receptor
IL-5R	interleukin 5 receptor
